# 眼附属器黏膜相关淋巴组织结外边缘区淋巴瘤的治疗及预后：单中心报道

**DOI:** 10.3760/cma.j.issn.0253-2727.2022.03.005

**Published:** 2022-03

**Authors:** 鑫 李, 进 叶, 磊 杨, 立强 魏, 佳 丛, 娜 姚, 晶 杨, 景文 王

**Affiliations:** 首都医科大学附属北京同仁医院血液科，北京 100730 Department of Hematology, Beijing Tongren Hospital, Capital Medical University, Beijing 100730, China

**Keywords:** 淋巴瘤，B细胞，边缘区, 预后, 放疗, 化疗, Lymphoma, B-cell, marginal zone, Prognosis, Radiotherapy, Chemotherapy

## Abstract

**目的:**

探讨局限于眼附属器黏膜相关淋巴组织结外边缘区淋巴瘤（OAML）患者应用不同初始治疗方案对长期预后的影响。

**方法:**

对2008年4月至2019年4月首都医科大学附属北京同仁医院血液科收治的109例初发OAML患者进行疗效评估及随访，对初始治疗方案与预后进行分析。

**结果:**

36例患者经手术完全切除病灶，73例术后存在残留病灶，其中37例选择观察等待，36例选择治疗，治疗方案包括局部放疗和系统性治疗（化疗、免疫化疗、放化疗联合等），选择系统性治疗患者未发生严重不良反应。中位随访时间61（10～142）个月，单眼受累患者5年、10年无进展生存（PFS）率分别为78.2％和76.0％，双眼受累患者5年、10年PFS率分别为64.4％和23.5％，单眼、双眼受累患者PFS的差异有统计学意义（*P*＝0.010）。残留病灶观察组患者和残留病灶治疗组患者5年PFS率分别为71.4％和90.1％，10年PFS率分别为63.5％和75.1％，两组患者PFS率的差异有统计学意义（*P*＝0.046）。OAML患者超过5年仍有疾病进展风险。

**结论:**

双眼受累OAML患者预后差，治疗可以降低复发或进展风险。系统性治疗可作为OAML患者的一线治疗选择之一。OAML需要长期随访。

## Abstract (en)

**Objective:**

This study aimed to see how different initial treatment regimens affected the long-term prognosis of patients with extranodal marginal zone mucosa-associated lymphoid tissue lymphoma confining to the ocular adnexal（OAML）.

**Methods:**

Between April 2008 and April 2019, 109 patients with initial mucosa-associated lymphoid tissue confining to ocular adnexal were evaluated and followed-up, and the prognosis of various initial treatment regimens were examined.

**Results:**

A total of 36 patients underwent complete surgical resection of the lesions, and 73 patients had residual lesions after surgery, of which 37 patients chose watchful waiting, and 36 patients chose treatment. The treatment regimen included local radiotherapy and systemic treatment（chemotherapy, immunochemotherapy, the combination of radiotherapy and chemotherapy, etc.）, and no serious toxic and side effects were observed in patients receiving systemic treatment. The median follow-up time was 61（10–142）months. The 5-year and 10-year progression-free survival（PFS）of monocular involvement patients were 78.2％ and 76.0％. The 5-year and 10-year PFS rates of patients with binocular involvement were 64.4% and 23.5%. There was significant diference in PFS between patients with monocular and binocular involvement（*P*＝0.010）. Patients who received additional treatment had higher PFS than those patients in the watchful waiting group（*P*＝0.046）. The 5-year PFS was 71.4％ and 90.1％ among patients in the watchful waiting group and those who received additional treatment, whereas the 10-year PFS was 63.5％ and 75.1％, respectively. Patients with OAML were still a risk of disease progression after 5 years.

**Conclusion:**

Patients with binocular involvement OAML at the start of the disease had a poor prognosis, but treatment could reduce the risk of recurrence/progression. Systemic therapy is one of the first-line treatment options for patients with OAML, who require long-term monitoring.

黏膜相关淋巴组织结外边缘区淋巴瘤（extranodal marginal zone lymphoma of mucosa-associated lymphoid tissue，MALT淋巴瘤）是一种低度恶性的B细胞非霍奇金淋巴瘤，是眼附属器淋巴瘤最常见的类型[1]–[2]。眼附属器MALT淋巴瘤（ocular adnexal MALT，OAML）指原发于眼附属器的MALT淋巴瘤，大多数为局限性病变，病灶多局限于眼附属器，包括结膜、眼睑、眼眶、泪腺等部位，少部分病例出现眼外部位受累[3]。

迄今，关于OAML的一线治疗尚无统一标准，考虑到MALT淋巴瘤的惰性病程，观察等待成为患者和医师认可的方案之一。对于局限型病灶，局部放射治疗是许多临床医师的优先选择，但放疗后白内障、干眼症等眼部并发症使部分患者对选择放疗产生顾虑，放射治疗的最佳放疗剂量仍在探讨中[4]。合并眼外病灶患者接受化疗或免疫治疗等系统性治疗有助于疾病控制，但如果病变局限于眼附属器，是否进行系统性治疗仍存在争议。本研究分析OAML患者应用不同初始治疗方案的疗效和安全性，初步探讨不同初始治疗方案对预后的影响，旨在为这部分患者治疗方案的选择提供指导。

## 病例与方法

1. 病例：纳入自2008年4月至2019年4月在北京同仁医院诊断的OAML患者109例，病变仅累及眼附属器，无淋巴结和其他器官受累。所有病例经眼部肿物切除或者活检送检病理组织，按照2016年WHO淋巴造血组织肿瘤分类标准明确诊断。入组患者完善全身体格检查、血液生化项目检查、影像学检查等，采用Ann Arbor分期和眼附属器淋巴瘤AJCC（American Joint Committee on Cancer）-TNM分期[5]；采用淋巴瘤国际预后指数（IPI）评分和MALT淋巴瘤国际预后指数（MALT-IPI）评分[6]。

2. 治疗方案：根据眼部肿物部位及浸润程度，患者选择肿物完全切除或者部分切除，36例患者经眼科手术完全切除肿物无残留病灶（无残留组），73例患者眼科术后仍有残留病灶，这部分患者根据病变累及情况、治疗意愿等因素分为两组，37例采取等待观察（残留观察组），36例进行治疗（残留治疗组），治疗方案包括局部放疗和系统性治疗。化疗方案具体为：COP方案（环磷酰胺 750 mg·m^−2^·d^−1^，第1天；长春地辛 4 mg/d，第1天；泼尼松60 mg·m^−2^·d^−1^，第1～5天；21 d为1个周期）、CHOP方案（环磷酰胺750 mg·m^−2^·d^−1^，第1天；吡柔比星 50 mg·m^−2^·d^−1^，第1天；长春地辛4 mg/d，第1天；泼尼松 60 mg·m^−2^·d^−1^，第1～5天；21 d为1个周期）、R-COP方案（利妥昔单抗 375 mg·m^−2^·d^−1^，第0天；环磷酰胺750 mg·m^−2^·d^−1^，第1天；长春地辛4 mg/d，第1天；泼尼松 60 mg·m^−2^·d^−1^，第1～5天；21 d为1个周期）、R-CHOP方案（利妥昔单抗 375 mg·m^−2^·d^−1^，第0天；环磷酰胺750 mg·m^−2^·d^−1^，第1天；吡柔比星 50 mg·m^−2^·d^−1^，第1天；长春地辛4 mg/d，第1天；泼尼松 60 mg·m^−2^·d^−1^，第1～5天；21 d为1个周期）、FND方案（氟达拉滨25 mg·m^−2^·d^−1^，第1～3天；米托蒽醌10 mg·m^−2^·d^−1^，第1天；地塞米松 20 mg/d，第1～5天）。

3. 疗效评价：按照Lugano缓解标准分为完全缓解（CR）、部分缓解（PR）、疾病进展（PD）、复发。在治疗结束后1个月及此后的每6个月进行疗效评价，采用全身体格检查，血液相关指标实验室检查及MRI、PET-CT等影像学评估。

4. 随访：以电话、门诊或查阅住院病历的方式进行随访，随访截止时间为2020年2月，中位随访时间61（10～142）个月。无进展生存（PFS）时间定义为自开始治疗到末次随访或任何原因导致疾病进展或死亡的时间。总生存（OS）时间定义为自开始治疗到末次随访或死亡的时间。

5. 统计学处理：采用SPSS 17.0软件进行统计学分析，计数资料用例数（百分比）表示，计量资料用中位数（范围）表示。组间比较采用Chi-square检验，生存分析采用Kaplan-Meier法，多因素分析采用Cox回归模型。*P*<0.05为差异有统计学意义。

## 结果

1. 临床特征：共纳入109例患者，男70例（64.2％），女39例（35.8％），中位发病年龄57（19～83）岁，≤60岁者68例（62.4％），>60岁者41例（37.6％）。2例（1.8％）患者伴LDH升高，1例（0.9％）患者伴B症状（消瘦）。美国东部肿瘤协作组（ECOG）评分0分、1分患者分别为98例（89.9％）和11例（10.1％）。Ann Arbor分期Ⅰ期、Ⅳ期（双眼受累）患者分别为88例（80.7％）和21例（19.3％）。IPI评分0～1分87例（79.8％），2～3分22例（20.2％）。MALT-IPI评分0分74例（67.9％），1分34例（31.2％），≥2分1例（0.9％）。常见症状包括眼部异物感、眼睑肿胀、无痛性肿块、眼球突出、视力下降等，中位症状持续时间9（1～90）个月。单眼受累88例（80.7％），双眼受累21例（19.3％）。TNM分期：T1N0M0 23例（21.1％），T2N0M0 53例（48.6％），T3N0M0 25例（22.9％），T4N0M0 8例（7.4％）。眼部病变部位以眼眶（T2）最常见，共53例（48.6％），其中单纯眼眶受累者41例（37.6％）；单纯累及结膜（T1）患者23例（21.1％）；单纯累及泪腺患者7例（6.4％）；单纯累及眼睑患者6例（5.5％）；多部位受累患者32例（29.4％）。8例T4患者中4例累及局部皮肤或皮下软组织，3例累及视神经，1例累及副鼻窦。

2. 疗效和随访：109例患者中36例通过手术完全切除病灶，余73例术后仍存在残留病灶，根据病变累及情况、治疗意愿等因素，37例等待观察，36例进行治疗。残留治疗组患者中13例患者接受局部放疗，剂量3 200～4 500 cGy。17例患者分别行COP方案（3例）、CHOP方案（6例）、R-COP方案（3例）、R-CHOP方案（3例）、FND方案（2例）治疗，21 d为1个周期，共4～6个周期。6例患者局部放疗联合化疗或免疫化疗，局部放射剂量2 600～3 600 cGy，化疗方案包括COP方案（1例）、CHOP方案（3例）、R-COP方案（1例）、R-CHOP方案（1例），21 d为1个周期，共4个周期。接受治疗患者中28例（84.8％）达CR，8例患者达PR。截至2020年2月，随访时间61（10～142）个月，27例（24.8％）患者PD或复发，持续缓解时间44（4～142）个月，其中4例患者超过5年发生PD。手术切除后无残留病灶患者中11例（30.6％）复发，持续缓解时间67（4～142）个月。残留观察组患者中11例（29.7％）发生PD，持续缓解时间34（5～118）个月。以上两组中复发/PD患者均表现为眼部原位复发或进展，其中3例同时出现眼外部位累及，分别为口腔黏膜、肘部皮肤、全身多处深部淋巴结。残留病灶接受治疗的患者中，2例CR患者出现眼眶原位复发，另外3例PR患者发生眼眶原位病情进展，持续缓解时间57（6～141）个月。109例患者中，27例（24.8％）患者发生PD或复发，持续缓解时间44（4～142）个月。放疗组（13例）患者中12例疗效为CR，1例疗效为PR，随访过程中1例PD。化疗±免疫治疗组（17例）患者中10例疗效为CR，7例疗效为PR，随访过程中2例复发，2例PD。放疗联合化疗组（6例）患者疗效均为CR，随访过程中无复发/PD。

3. 安全性：19例接受放射治疗患者放射剂量2 600～4 500 cGy，放疗后10例患者发生结膜炎，7例出现局部皮肤肿胀，结膜炎及皮肤损伤在放疗半年内陆续恢复。随访期内所有患者均未行白内障手术，3例治疗结束半年后诊断干眼症，发生率15.8％。23例患者接受化疗，不良反应包括Ⅰ～Ⅱ度消化道反应9例、Ⅰ～Ⅱ度骨髓抑制6例、Ⅱ度肝功能异常1例、Ⅲ度骨髓抑制1例。化疗后仅1例患者合并呼吸道感染，发生率仅4.3％。

4. 生存分析：109例OAML患者5年、10年OS率均为100.0％，5年、10年PFS率分别为75.6％、67.1％。无残留患者5年、10年PFS率分别为69.0％、65.3％。残留观察和残留治疗患者的5年PFS率分别为71.4％和90.1％，10年PFS率分别为63.5％和75.1％。对三组患者进行生存分析，残留治疗和残留观察患者PFS率的差异有统计学意义（*P*＝0.046）（图1）。治疗患者中放疗、化疗±免疫化疗、放疗联合化疗患者的5年PFS率分别为90.0％、86.3％、100.0％，三组PFS率的差异无统计学意义（图2）。进一步分析临床特征和预后之间的相关性，发现基线IPI评分、单/双眼受累与PFS相关。单眼受累患者的5年、10年PFS率分别为78.2％、76.0％，双眼受累患者的5年、10年PFS率分别为64.4％、23.5％，两组PFS的差异有统计学意义（*P*＝0.010）（图3）。IPI评分0～1分组5年、10年PFS率分别为78％、75.8％，2～3分组5年、10年PFS率分别为66.2％和24.1％，两组PFS的差异有统计学意义（*P*＝0.017）（图4）。不同年龄、MALT-IPI评分和TNM分期患者的PFS差异无统计学意义。将年龄、单/双眼受累、IPI评分和治疗方案纳入多因素分析，以上因素均无独立预后意义（*P*值均>0.05）。

**图1:**
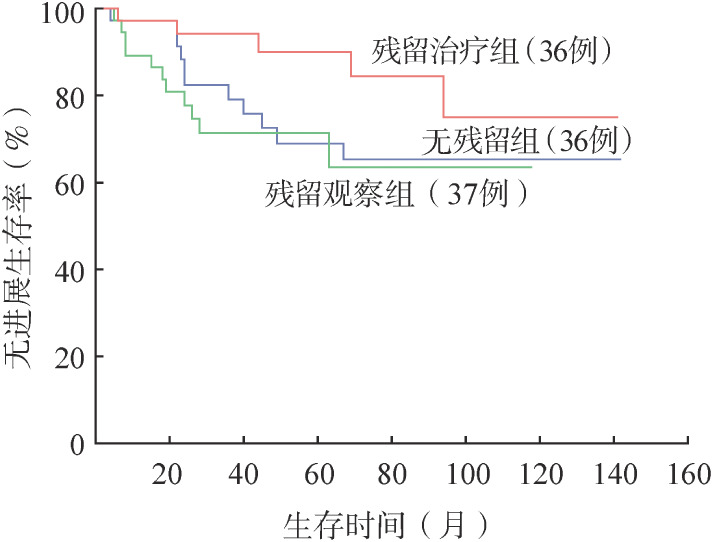
无残留组、残留观察组、残留治疗组OAML患者的无进展生存曲线 OAML：眼附属器黏膜相关淋巴组织结外边缘区淋巴瘤；残留治疗组与残留观察组比较*P*＝0.046；残留治疗组与无残留组比较*P*＝0.136

**图2:**
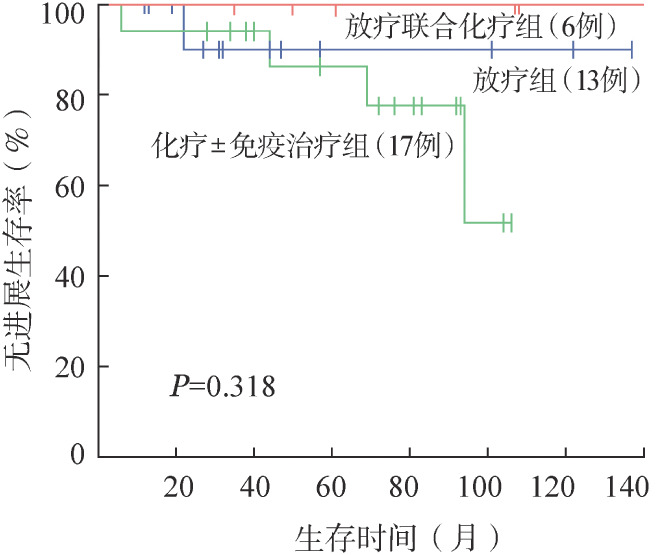
放疗组、化疗±免疫治疗组、放疗联合化疗组OAML患者的无进展生存曲线 OAML：眼附属器黏膜相关淋巴组织结外边缘区淋巴瘤

**图3:**
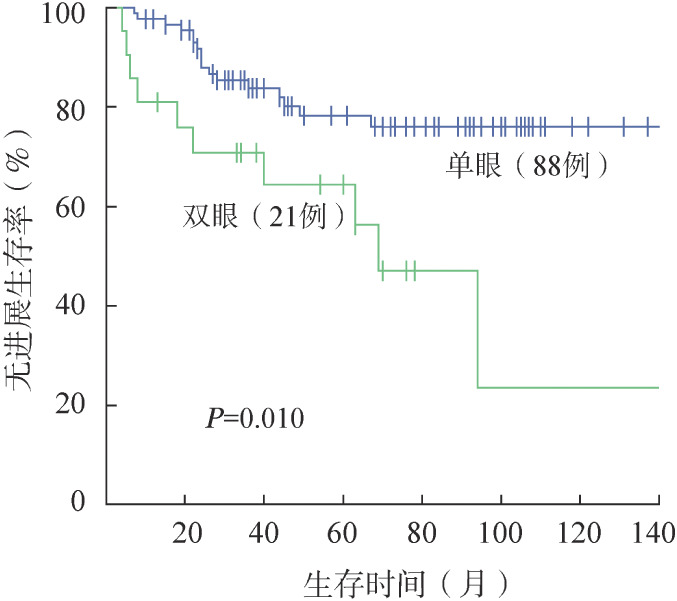
单、双眼受累OAML患者的无进展生存曲线 OAML：眼附属器黏膜相关淋巴组织结外边缘区淋巴瘤

**图4:**
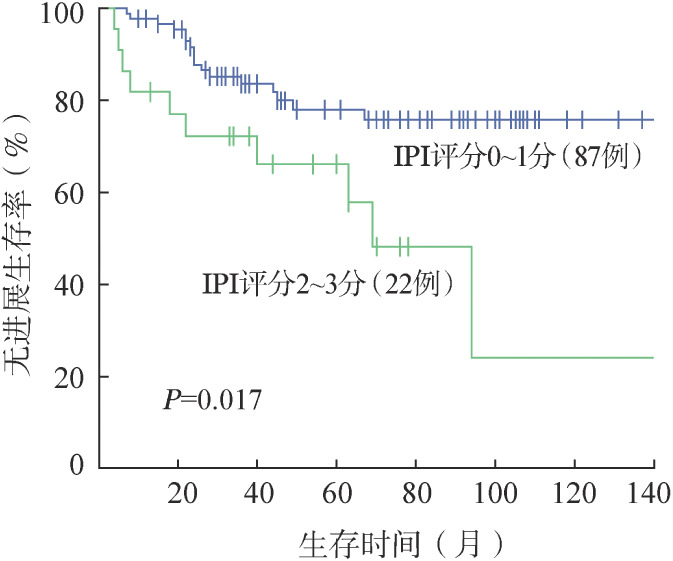
IPI评分0～1分和2～3分OAML患者的无进展生存曲线 OAML：眼附属器黏膜相关淋巴组织结外边缘区淋巴瘤

## 讨论

眼附属器淋巴瘤是眼附属器的恶性肿瘤，在眼肿瘤中约占55％，MALT淋巴瘤是眼附属器淋巴瘤中最常见的病理类型，占38％～98％[1]–[3],[7]–[8]。近年来OAML发病率逐年增加，且亚洲国家的发病率显著高于西方国家[9]–[10]。OAML多发生于男性，发病的中位年龄波动于41～72岁[2]–[3],[11]，本研究中男性比例达64.2％，中位发病年龄57岁。入组患者仅1例伴B症状，2例血清LDH水平升高。据文献报道，局限于眼附属器的MALT淋巴瘤患者不伴随B症状，LDH升高比例低于10％[2]，这与MALT淋巴瘤的惰性临床病程相符。OAML眼部病变多为单眼受累，最常累及部位是眼眶，其次为结膜、眼睑、泪腺，局部病变广泛可发生复合病变，同时累及眼附属器多个部位，文献报道眼眶受累率超过40％，结膜受累率35％～40％，眼睑受累率10％～15％，泪腺受累率低于10％[1]–[3]。在本研究中，OAML患者眼眶受累率48.6％，单眼受累者占80.7％，多部位受累者占29.4％。

目前OAML治疗方案尚无统一标准，手术切除不仅能够获取病理标本明确诊断而且能达到治疗的目的。手术切除后仍存在残留病灶的患者需根据病变累及情况、对器官功能的影响、患者意愿等因素考虑观察或予其他治疗。无论选择放疗、化疗还是免疫联合治疗等，初始治疗有效率可达95％～100％[2]–[3],[12]，但仍有局部及远处复发风险，复发率高达14.4％～48.0％ [13]–[17]。Nam等[4]的研究纳入198例OAML患者，对病变局限于眼附属器患者进行放疗，放疗剂量30（20～45）Gy，治疗有效率达96.15％，复发率14.14％。Oh等[13]的研究纳入84例双眼同时受累的OAML患者进行双眼放疗，单眼放疗剂量27（20～40）Gy，CR率80.9％，PR率16.7％，10年PFS率和OS率分别为79.8％和91.1％，随访期间11例患者进行了白内障手术。韩国学者将R-CHOP/R-CVP作为局限于眼附属器的MALT淋巴瘤的一线治疗，总有效率100.0％，33例患者中仅3例复发，4年PFS率达90.3％[18]。本研究中36例进行治疗患者的CR率84.8％，PR率15.2％，总有效率100％，随访过程中5例患者发生病情进展，复发/PD率13.9％。本研究对术后存在残留病灶患者进行随访及生存分析，发现残留治疗患者的PFS率高于残留观察患者，提示对于OAML患者，手术以外的干预能够降低疾病进展风险，获得更长的无病生存期。Jeon等[17]将177例T1-2N0M0的OAML患者分为放疗、联合治疗、手术切除三组，其中联合治疗的方案包括CHOP/CVP±利妥昔单抗±放疗，三组的复发率分别为8.7％、7.0％、20.0％，放疗或联合治疗患者的复发率明显低于手术患者。

既往文献报道，病灶局限患者进行局部放疗即可获得较理想的预后，系统性治疗如化疗、免疫治疗、放化疗联合等更多应用在合并远处转移的病例。但放疗引起的干眼症、白内障等不良反应对患者日常生活的影响逐渐受到重视。本研究放疗后干眼症的发生率为15.8％。韩国一项关于结膜OAML放疗的研究显示，79例患者放疗后干眼症、白内障、眼痛发生率分别为26.6％、6.3％、5.1％[19]。日本学者的研究显示，46例眼附属器淋巴瘤患者放疗后高达30％的患者接受了白内障手术[20]。因此，我们认为眼附属器局部放疗引起的不可逆损伤可能降低部分OAML患者的生活质量。

本研究中入组病例病变均局限于眼附属器，将化疗、免疫治疗、放化疗联合等系统性治疗作为一线治疗选择的患者可获得有效的疾病控制，治疗过程中未发生严重不良反应，仅出现轻度骨髓抑制、消化道反应等，短期内即可恢复。Kim等[18]研究发现应用R-CVP方案治疗非结膜及双眼受累的局限期OAML患者，4年PFS率和OS率分别达90.3％和100％，所有患者均顺利完成6个周期R-CVP方案联合2个周期利妥昔单抗单药治疗，无需剂量调整，治疗期间仅三分之一患者出现中性粒细胞减少及末梢神经炎，不良反应可耐受。另一项关于Ⅰ～Ⅳ期OAML患者的前瞻性研究显示，放化疗联合能很好地控制疾病，且患者可耐受放化疗的不良反应 [21]。因此我们认为OAML患者接受系统性治疗引起的不良反应是可控的、可逆的，系统性治疗可以作为局限期OAML患者的一线治疗选择之一。

本研究结果显示IPI低危组（0～1分）患者的PFS率明显高于中危组（2～3分），似乎提示IPI评分对于局限期OAML患者的预后有一定预测意义。但结合本研究入组病例的特点，IPI评分主要与双眼受累相关，即双眼受累患者因Ann Arbor分期Ⅳ期、结外受累部位数≥2个被归为IPI中危组。研究中未发现MALT-IPI评分对OAML患者的预后有预测意义，可能的原因是MALT-IPI是IELSG-19临床试验总结得出的针对任何部位MALT淋巴瘤的预后评估体系，其中胃肠MALT淋巴瘤达42.6％，对于OAML仍有一定局限性。多项研究指出OAML双眼受累的病例有更高的复发风险[22]–[23]。本研究也显示单眼受累患者PFS率高于双眼受累患者。考虑到单眼和双眼受累患者的预后存在差别，应该分为不同疾病阶段，故本文将单眼受累患者纳入Ann Arbor Ⅰ期，双眼受累患者纳入Ann Arbor Ⅳ期。因此，关于OAML如何更合理地进行预后分组、除双眼受累外是否还有其他有意义的预后指标仍需要扩大样本量、延长随访时间、多中心协作的临床研究进一步明确，以得出适用于眼附属器淋巴瘤的独特预后评估体系。

因OAML发病部位的特殊性及逐年增加的发病率，近年来得到越来越多的关注。本研究发现，OAML患者手术治疗后如有残留病灶，适当治疗可降低复发风险，延长PFS时间。系统性治疗可避免放疗引起的干眼症等不良反应，可作为OAML患者的一线治疗选择之一。初治时双眼受累患者在随访期间需警惕疾病进展。
